# Breeding chronology and social interactions affect ungulate foraging behavior at a concentrated food resource

**DOI:** 10.1371/journal.pone.0178477

**Published:** 2017-06-07

**Authors:** David B. Stone, Michael J. Cherry, James A. Martin, Bradley S. Cohen, Karl V. Miller

**Affiliations:** 1 Warnell School of Forestry and Natural Resources, University of Georgia, Athens, Georgia, United States of America; 2 Department of Fish and Wildlife Conservation, Virginia Tech, Blacksburg, Virginia, United States of America; 3 Savannah River Ecology Lab, University of Georgia, Aiken, South Carolina, United States of America; Université de Sherbrooke, CANADA

## Abstract

Prey species must balance predator avoidance behavior with other essential activities including foraging, breeding, and social interactions. Anti-predator behaviors such as vigilance can impede resource acquisition rates by altering foraging behavior. However, in addition to predation risk, foraging behavior may also be affected by socio-sexual factors including breeding chronology and social interactions. Therefore, we investigated how time-of-day, distance-to-forest, group size, social interactions (presence of different sex-age class), and breeding chronology (pre-breeding, breeding, post-breeding seasons) affected probability of feeding (hereafter: feeding) for different sex and age-classes (mature males, immature males, adult females, and juveniles) of white-tailed deer at feed sites. We developed a set of candidate models consisting of social, habitat, reproductive, and abiotic factors and combinations of these factors. We then used generalized linear mixed models (GLMMs) to estimate the probability of feeding and used model averaging of competing models for multimodel inference. Each adult sex-age class’ feeding was influenced by breeding chronology. Juveniles were more likely to be feeding than adults in all seasons. Feeding increased with group size for all sex-age classes. The presence of a mature male negatively influenced the feeding of immature males and juveniles were more likely to be feeding when an adult female was present. Feeding decreased with increasing distance-to-forest for mature males but not for other sex-age classes. Our results indicate that each sex-age class modulates vigilance levels in response to socio-sexual factors according to the unique pressures placed upon them by their reproductive status and social rank.

## Introduction

Prey species must balance predator avoidance with foraging, reproductive behaviors, and social interactions [1,2]. These decisions manifest in anti-predator behaviors which can hinder resource acquisition rates [1,3,4]. Because predation risk varies temporally and spatially and is perceived by animals on both fine- and coarse-temporal and spatial scales [5,6], prey can modify their immediate behavioral state, social cohesion, or selection patterns to decrease predation risk. These responses include changing group size [7], selecting safer areas [8], foraging closer to escape cover [9,10], reducing foraging activity [1], shifting foraging activity times [11], and increasing vigilance [12]. Vigilance is a common metric for measuring anti-predator behavior because it is directly related to resource acquisition rates that affect prey fitness [13]. However, in addition to predation risk, socio-sexual factors including sex [10], social rank [3], presence of conspecifics [14], and reproductive status [15] may also modulate vigilance behaviors thereby affecting foraging behavior.

In ungulates, sex-specific differences in vigilance are species-dependent. The demands of neonate provisioning and protection increase vigilance of females for some ungulate species, such as elk [*Cervus elaphus;* [10]] and moose [*Alces alces*; [16]]. Yet, in other ungulate species including springbok [*Antidorcas marsupialis*; [17]] and waterbuck [*Kobus defassa*; [18]], males may be more vigilant. Moreover, some ungulates, such as impala [*Aepyceros melampus*; [18]], show no sex-specific differences in vigilance levels. Furthermore, sex-specific vigilance depends on the animal’s reproductive status, nutritional condition, and vulnerability to predation [10,14].

When foraging in groups, an individual’s vulnerability to predation is reduced due to dilution and detection effects [19–21]. However, differential sex-specific group size effects may exist in some ungulates [13,14,22]. For example, Zheng et al. (2013] reported that individual vigilance decreased with increasing group size in all-female groups of Père David’s deer (*Elaphurus davidianus*) but not in all-male or mixed-sex groups. In mixed-sex groups, females did not lower their vigilance, perhaps because vigilance was not directed at predators but rather at male conspecifics [22].

Social rank can play an important role when vigilance is directed at conspecifics [15]. In social ungulates that form aggregations governed by dominance hierarchies, subordinates direct their vigilance at more dominant animals to avoid aggressive interactions [23]. Conversely, dominant males may show higher vigilance levels than subordinate males when defending a harem from potential competitors [14]. Agonistic interactions are energetically expensive [24] and, when coupled with reduced resource acquisition rates while vigilant, can create a deficit in the energy-predator avoidance balance that must be achieved for prey animals to maximize fitness.

Ungulates exhibit variable trends in vigilance during the breeding season depending on the breeding ecology of the species and the social rank of the individual. In harem breeders such as elk, males are more vigilant during the breeding season than the non-breeding season [14]. In contrast, male ungulates employing a territorial breeding strategy, such as Przewalski's gazelle [*Procapra przewalskii;* [15]], may invest more heavily in foraging than vigilance during the breeding season, whereas increased vigilance by non-breeding males and females may result from avoidance of aggression of dominant, breeding males [15].

Little is known about white-tailed deer (*Odocoileus virginianus*) vigilance and how social interactions and rank, breeding chronology, and other factors affect their anti-predator behaviors. White-tailed deer typically are sexually segregated throughout most of the year [25] with males forming loose aggregations consisting of different age-classes outside of the breeding season [26,27]. Older and larger males tend to be dominant over immature males and adult females [28,29]. Among mature males, dominance is more correlated with body mass than age [28]. In contrast, dominance among females is correlated with both increasing age [28] and body mass [30]. Nevertheless, large, mature males hold the highest social rank followed by adult females, immature males, and juveniles.

During the breeding season, non-estrous females may be subjected to harassment by rutting males. The duration of behavioral estrus in white-tailed deer is approximately 24 h [31] but may last >48 h [32]. Consequently, during much of the breeding season, females tend to avoid rutting males. Additionally, subordinate males strive to avoid agonistic interactions with dominant males. Therefore, investigating foraging behavior prior to, during, and after the breeding season may offer insight on how vigilance changes in response to changes in socio-sexual factors such as breeding chronology and presence of conspecifics, in addition to perceived predation risk.

There is an inverse relationship between foraging and vigilance whereby the cost of vigilance is a decrease in foraging. Therefore, in order to better understand foraging-vigilance tradeoffs, we investigated factors influencing foraging behavior, specifically the probability of feeding while at a concentrated food resource. We hypothesized that each sex-age class would show unique responses to reproductive (phase of breeding season), social (presence of different sex-age class, group size), predation risk (distance-to-forest), and abiotic (time-of-day) cues. We predicted males would exhibit stronger responses to socio-sexual factors than adult females and juveniles. We predicted adult females would be most sensitive to predation risk during the pre-breeding season when their offspring were more vulnerable to predation [13,16]. We predicted feeding would be greater during diurnal periods [33] but decrease as distance-to-forest increased because of greater perceived risk associated with open areas. We predicted that all sex-age classes would perceive less individual predation risk when foraging in larger groups [7,33] and consequently increase feeding. Finally, we predicted subordinate sex-age classes would decrease feeding when in the presence of a mature male as vigilance would likely be directed at avoiding agonistic interactions with the more dominant sex-age class [13,15].

## Material and methods

### Ethics statement

All applicable international, national, and/or institutional guidelines for the care and use of animals were followed and approved by the University of Georgia Institutional Animal Care and Use Committee (IACUC #A2012 06-007-Y3-A2).

### Study area

We conducted this study on a privately-owned, 1,619 ha property in Harris County, GA, USA (32.8023°N, −84.9049°W). The landowner (Joe Rogers) granted us permission to conduct all research activities on his property. Elevations ranged from 200–275m. Habitat types on the study site included a mixture of pine, pine-hardwoods, hardwood drainages, and open areas. Pine stands made up approximately 983 ha (61%) of the land cover and were comprised primarily of loblolly (*Pinus taeda*) and shortleaf pine (*P*. *echinata*). Hardwood stands constituted approximately 582 ha (36%) of the study site and were dominated by oak (*Quercus* spp.), hickory (*Carya* spp.), tulip-poplar (*Liriodendron tulipifera*), and sweetgum (*Liquidambar styraciflua*). Open areas included pasture, fallow fields, row crops, and cultivated wildlife openings. Recreational white-tailed deer hunting was allowed on the property from the second Saturday in September to 15 January. The property received minimal hunting pressure and approximately 10 (<1 deer per 160 ha) white-tailed deer were harvested annually. Non-human predators on the site included bobcats (*Lynx rufus*) and coyotes (*Canis latrans*).

### Experimental design

We established 22 feeding sites in a variety of vegetation types across the study site and used shelled corn as an attractant. Feed was presented via trough-style feeders (trough), barrel feeders (barrel), or placed directly on the ground (ground). Sites were established >2 weeks prior to data collection to allow deer to acclimate to the feeders. Once established, feed was maintained for the duration of the study.

From 13 September − 3 January in 2013 and 2014, we used infrared cameras (Reconyx Hyperfire 550, Holmen, WI, USA) to observe foraging behavior at the feed sites. We analyzed camera trap data for three days (Monday, Tuesday, and Wednesday) of each week for 16 weeks. Cameras were mounted to a tree or post 3–4 m from the feed site and approximately 75 cm from ground level. Cameras were triggered by motion and programmed to collect photographs 24hrs per day, with a 5-minute delay between successive photographs. We checked camera sites at least weekly to replenish feed and to replace camera batteries as needed. Typically, feeding sites were replenished three times per week but during the breeding season, deer used the sites less intensively, therefore requiring less maintenance.

We assigned each observed deer to a sex-age class [mature male (≥3.5 years-old), immature male (1.5–2.5 years-old), adult female (≥1.5 years-old), juvenile (<1.5 years-old)] based on antler and body morphology [34], and recorded the time and date of the photograph. We assigned the photograph to 1 of 3 seasons (relative to breeding season) based on conception data from the study site [35]: 1) Pre-breeding—weeks 1–6 (13 September − 25 October); 2) Breeding—weeks 7–11 (26 October − 27 November); and 3) Post-breeding—weeks 12–16 (28 November − 3 January). To quantify visitation rates, we calculated mean images per site per week for the pre-breeding, breeding, and post-breeding seasons for each sex-age class. We classified time-of-day as either diurnal (30 minutes prior to sunrise − 30 minutes after sunset) or nocturnal (>30 minutes after sunset − >30 minutes prior to sunrise) based on the median sunrise-sunset for that week. We measured the distance from the feed site to the nearest forest in all four cardinal directions using ArcMap 10.1 [36] and calculated the mean. This method more accurately characterized the openness of the habitat in which the feeding site was located than distance-to-forest in one direction only, as all feeding sites, including those that were located in open areas, were within 43m from the nearest forest edge. Distance-to-forest was standardized by calculating a *z*-score for the distance variable for each feeding site.

We chose to model foraging behavior and not vigilance because ungulates can process food while in a vigilant posture. Therefore, foraging is more directly related to resource acquisition than vigilance. We characterized foraging behavior as “actively feeding” if the deer was consuming feed either directly from the ground or feeder. Because all types of feeders did not necessarily require the deer to have its head down in a typical foraging posture to access feed, we did not use head position alone to categorize behavioral state as done in previous studies [13,33]. If the deer was not actively consuming feed from the ground or feeder, we characterized the behavioral state as “not actively feeding”. We attempted to minimize variation in behavioral interpretation by using a single observer (DBS).

We developed a set of candidate models consisting of variables known to affect foraging behavior including breeding chronology (pre-breeding, breeding, and post-breeding seasons), social interactions (presence of different sex-age class, group size), predation risk (distance-to-forest), and abiotic factors (time-of-day; Table 1). The binomial response variable was actively feeding (1) or not actively feeding (0). We then fitted generalized linear mixed models (GLMMs) to estimate the probability of feeding and treated feeding site as a random variable. We included feeding site as a random variable to account for the non-independence of our observations from the same feeding site.

**Table 1:** Candidate models used to predict the probability of feeding for mature male (≥3.5 years-old), immature male (≤2.5 years-old), adult female (≥1.5 years-old), and juvenile (<1.5 years-old) white-tailed deer at feeding sites in Harris County, GA, USA (September-January 2013 and 2014).

**Mature males**
1) Distance-to-forest*Time-of-day + Season*Time-of-day + Immature male*Season + Adult Female + Juvenile + Group Size 2) Distance-to-forest*Time-of-day + Immature male*Season + Adult Female + Juvenile + Group Size 3) Season*Time-of-day + Immature male*Season + Adult Female + Juvenile + Group Size 4) Immature male*Season + Adult Female + Juvenile + Group Size 5) Distance-to-forest*Time-of-day + Season*Time-of-day 6) Season*Time-of-day 7) Distance-to-forest*Time-of-day 8) Null
**Immature males**
1) Distance-to-forest*Time-of-day + Season*Time-of-day + Mature male*Season + Adult Female + Juvenile + Group Size 2) Distance-to-forest*Time-of-day + Mature male*Season + Adult Female + Juvenile + Group Size 3) Season*Time-of-day + Mature male*Season + Adult Female + Juvenile + Group Size 4) Mature male*Season + Adult Female + Juvenile + Group Size 5) Distance-to-forest*Time-of-day + Season*Time-of-day 6) Season*Time-of-day 7) Distance-to-forest*Time-of-day 8) Null
**Adult females**
1) Distance-to-forest*Time-of-day + Season*Time-of-day + Mature male + Immature Male + Juvenile*Season + Group Size 2) Distance-to-forest*Time-of-day + Mature male + Immature Male + Juvenile*Season + Group Size 3) Season*Time-of-day + Mature male + Immature Male + Juvenile*Season + Group Size 4) Mature male*Season + Adult Female + Juvenile + Group Size 5) Distance-to-forest*Time-of-day + Season*Time-of-day 6) Season*Time-of-day 7) Distance-to-forest*Time-of-day 8) Null
**Juveniles**
1) Distance-to-forest*Time-of-day + Season*Time-of-day + Mature male+ Immature Male + Adult Female*Season + Group Size 2) Distance-to-forest*Time-of-day + Mature male + Immature Male + Adult Female*Season + Group Size 3) Season*Time-of-day + Mature male+ Immature Male + Adult Female*Season + Group Size 4) Mature male*Season + Adult Female + Adult Female*Season + Group Size 5) Distance-to-forest*Time-of-day + Season*Time-of-day 6) Season*Time-of-day 7) Distance-to-forest*Time-of-day 8) Null

Predictor variables include standardized distance-to-forest, time-of-day (day = 1, night = 0), season (pre-breeding, breeding, post-breeding), presence of a mature male, presence of an immature male, presence of an adult female, presence of a juvenile, group size, year, feeder type, and feeding site. Each model included year, feeder type, and feeding site. Feeding site was treated as a random effect.

We first tested the residuals from the global model for serial autocorrelation in photographs for each sex-age class using the ‘acf’ function in Program R [37]. All auto-correlation values were <0.1 indicating that serial auto-correlation was not an issue. {{246 Burnham, Kenneth P 2003}}We used Akaike’s Information Criteria adjusted for small sample size (AICc) and considered any model with a ΔAICc value of ≤2 as a competing model [38]. We used model averaging for competing models and based our inferences on coefficients with 95% confidence intervals (CI) that did not include zero. We used the full average estimates for competing models [38].

We included the interactions between distance-to-forest and time-of-day as well as distance-to-forest and season because perceived predation risk may change according to the diel cycle or as physiological condition changes relative to the breeding season. For the mature and immature male models, we included an interaction between their presence and season as the frequency of agonistic interactions between males is influenced by the phase of the breeding season [26], and these interactions may influence resource acquisition rates. Additionally, for the adult female and juvenile models, we included interaction terms for their presence in the respective models and season as females may decrease feeding rates when juveniles are present [13]. To control for potential differences in feeding rates influenced by year and feeder type, we included year and feeder type in each of our candidate models. We used Program R 3.1.2 for all statistical analyses [37].

## Results

Camera trapping effort was 375, 411, and 402 camera days for the pre-breeding, breeding, and post-breeding seasons, respectively. We collected 6,994 photographs containing images of 8,469 white-tailed deer for which we could assign a sex-age class. We recorded a total of 2,078 mature male, 2,479 immature male, 2,225 adult female, and 1,687 juvenile images.

Mean photos per site per week (hereafter: photographic occurrences;$\bar{x}\;\pm \;se$) were greatest during the post-breeding season for mature males (8.23 ± 0.89), immature males (11.39 ± 1.01), and juveniles (6.63 ± 0.65). Mean photographic occurrences were fewest during the breeding season for mature males (2.53 ± 0.37) and immature males (2.38 ± 0.32). In contrast, adult female photographic occurrences were similar among the pre-breeding (7.81 ± 1.38), breeding (5.71 ± 0.74), and post-breeding (6.78 ± 0.59) seasons.

Because of very few (*n* = 6) observations of mature males and adult females occurring in the same photograph, we failed to obtain convergence for the adult female and mature male models when we included their presence in the respective models. We also failed to obtain convergence for the mature male and immature male models when we included the seasonal interaction. Therefore, we removed those variables from the respective models.

**Fig 1:**
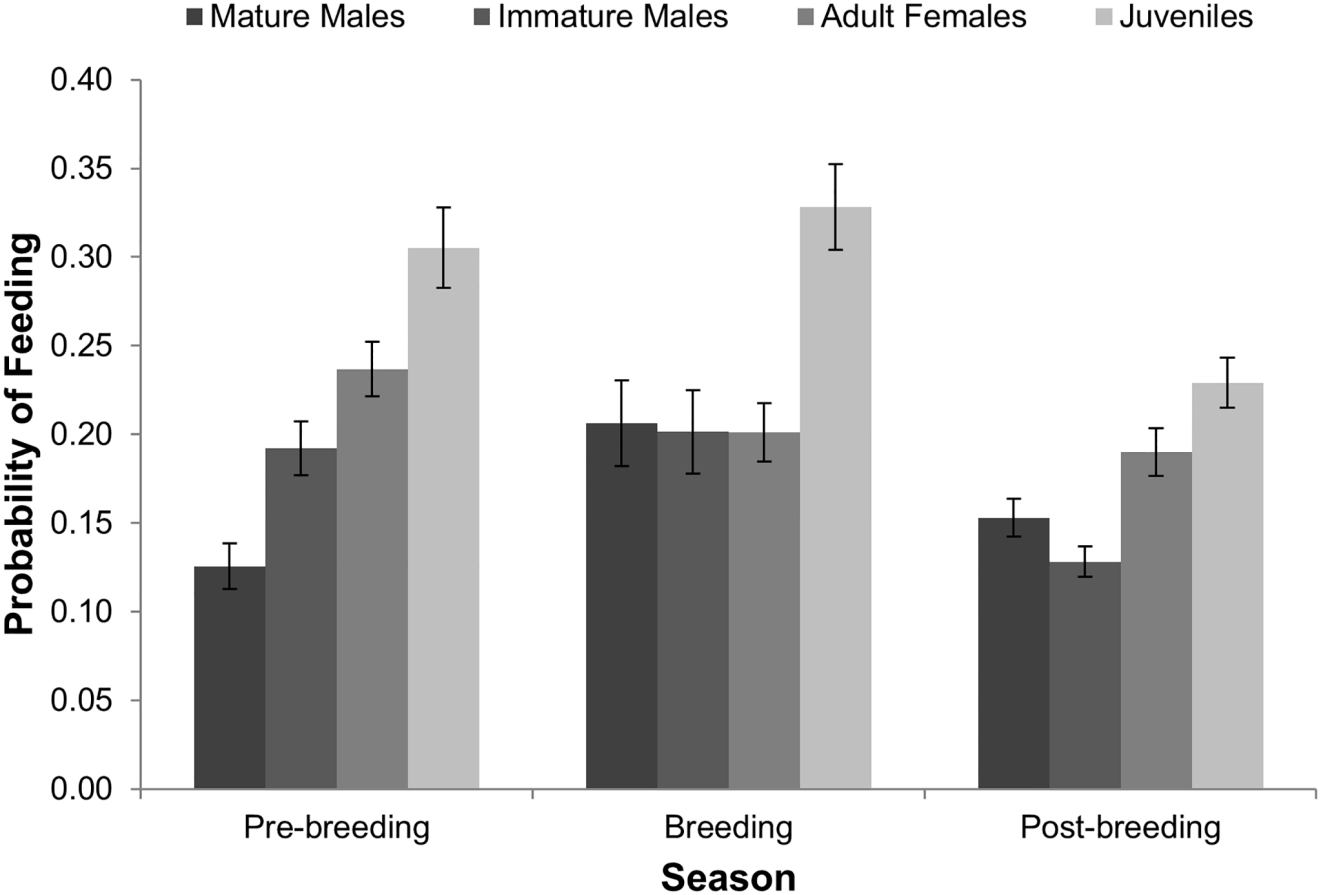
Influence of breeding chronology on proportion of time-spent feeding for white-tailed deer. Each sex-age class showed unique response to breeding chronology.

Mature male feeding was best explained by model 1 that included distance-to-forest *x* time-of-day interaction, season *x* time-of-day interaction, presence of an immature male, presence of an adult female, presence of a juvenile, group size, year, feeder type, and feeding site (Table 2). Neither the distance-to-forest *x* time-of-day interaction nor the season *x* time-of-day interaction was significant. Feeding increased with increasing group size, decreased with increasing distance-to-forest, and was greatest during the breeding season (Table 3, Fig 1). The variance estimate for feeding site was 0.113.

**Table 2:** Model selection results for models used to predict the probability of feeding for mature male (≥3.5 years-old), immature male (≤2.5 years-old), adult female (≥1.5 years-old), and juvenile (<1.5 years-old) white-tailed deer at feeding sites in Harris County, GA, USA (September-January 2013 and 2014). Models presented received the most support of our candidate models (ΔAICc <2).

Sex-age Class	Model^a^	K	AICc	ΔAICc	AIC Weight	Log-likelihood
**Mature**	1) D*T + S*T + IM + AF +J + GS	15	1672.64	0.00	1.0	-821.20
**Male**						
**Immature**	1) D*T + S*T + MM + AF + J + GS	16	2104.97	0.00	0.47	-1036.38
**Male**	3) S*T + MM + AF + J + GS	14	2106.33	1.35	0.24	-1039.08
**Adult**	1) D*T + S*T + IM + J*S + GS	17	2233.03	0.00	0.36	-1099.37
**Female**	3) S*T + IM + J*S + GS	15	2233.22	0.19	0.33	-1101.50
	4) IM + J*S + GS	12	2234.04	1.02	0.22	-1104.95
**Juvenile**	4) IM + AF*S + GS	12	1905.68	0.00	0.77	-940.75

^a^ Predictor variables include standardized distance-to-forest (D), time-of-day (T), season (S), presence of a mature male (MM), presence of an immature male (IM), presence of an adult female (AF), presence of a juvenile (J), and group size (GS). Year, feeder type, and feeding site were included in all models. Feeding site was treated as a random effect.

**Table 3:** Parameter estimates for generalized linear mixed model predicting the probability of feeding for mature male (≥3.5 years-old) white-tailed deer in Harris County, GA, USA, September–January, 2013–2014.

	*β*	SE	*z* value	Lower CI	Upper CI
**Intercept**	-2.00	0.38	-5.25	-2.752	-1.256*
**D**^a^	-0.65	0.18	-3.67	-0.993	-0.301*
**T**^b^	0.05	0.33	0.14	-0.599	0.692
**Post-breeding**^c^	-0.49	0.21	-2.32	-0.908	-0.076*
**Pre-breeding**^c^	-0.71	0.23	-3.11	-1.157	-0.261*
**IM**^d^	-0.29	0.33	-0.89	-0.930	0.348
**J**^e^	-0.56	0.57	-0.97	-1.678	0.566
**Y**^f^	-1.18	0.20	-5.94	-1.568	-0.790*
**GS**^g^	1.06	0.23	4.62	0.610	1.509*
**FT**^h^^i^	0.57	0.42	1.37	-0.245	1.387
**FT**^h^^j^	-0.28	0.35	-0.80	-0.961	0.405
**D x T**	0.24	0.20	1.19	-0.156	0.643
**T x Post-breeding**	0.11	0.38	0.29	-0.628	0.844
**T x Pre-breeding**	-0.21	0.52	-0.40	-1.231	0.810

* Indicates 95% confidence interval does not include zero.

^a^ Standardized distance-to-forest

^b^ Time-of-day (1 = diurnal, 0 = nocturnal)

^c^ Compared to reference class: breeding season

^d^ Presence of an immature male

^e^ Presence of a juvenile

^f^ Year

^g^ Group size

^h^ Feeder type, compared to reference class: barrel feeder

^i^ Feeder type = ground

^j^ Feeder type = trough

Immature male feeding was best explained by models 1 and 3 (Table 2). The best-fitting models included distance-to-forest *x* time-of-day interaction, season *x* time-of-day interaction, presence of a mature male, presence of an adult female, presence of a juvenile, group size, year, feeder type, and feeding site. Neither the distance-to-forest *x* time-of-day interaction nor the season *x* time-of-day interaction was significant. Feeding increased with increasing group size, decreased when a mature male was present, and was greater during the pre-breeding and breeding seasons than the post-breeding season (Table 4, Fig 1). The variance estimate for feeding site was 0.034.

**Table 4:** Parameter estimates for generalized linear mixed model predicting the probability of feeding for immature male (≤2.5 years-old) white-tailed deer in Harris County, GA, USA, September−January, 2013−2014.

	*β*	SE	*z* value	Lower CI	Upper CI
**Intercept**	-2.04	0.29	6.95	-2.621	-1.469*
**D**^a^	-0.01	0.09	0.09	-0.193	0.176
**T**^b^	0.01	0.35	0.03	-0.668	0.687
**Post-breeding**^c^	-0.45	0.20	2.24	-0.850	-0.058*
**Pre-breeding**^c^	0.04	0.22	0.18	-0.384	0.460
**MM**^d^	-0.88	0.32	2.70	-1.510	-0.241*
**AF**^e^	-0.25	0.55	0.46	-1.336	0.833
**J**^f^	-0.25	0.43	0.57	-1.094	0.603
**Y**^g^	0.01	0.15	0.06	-0.280	0.299
**GS**^h^	0.45	0.17	2.70	0.124	0.783*
**FT**^i^^j^	0.56	0.25	2.20	0.062	1.059*
**FT**^i^^k^	-0.19	0.21	0.90	-0.599	0.223
**D x T**	-0.23	0.21	1.09	-0.636	0.181
**T x Post-breeding**	0.34	0.38	0.88	-0.413	1.088
**T x Pre-breeding**	-0.11	0.43	0.25	-0.956	0.739

* Indicates 95% confidence interval does not include zero

^a^ Standardized distance-to-forest

^b^ Time-of-day (1 = diurnal, 0 = nocturnal)

^c^ Compared to reference class: breeding season

^d^ Presence of a mature male

^e^ Presence of an adult female

^f^ Presence of a juvenile

^g^ Year

^h^ Group Size

^i^ Feeder type, compared to reference class: barrel feeder

^j^ Feeder type = ground

^k^ Feeder type = trough

Adult female feeding was best explained by models 1, 3, and 4 (Table 2). The best-fitting models included distance-to-forest *x* time-of-day interaction, season *x* time-of-day interaction, presence of an immature male, presence of a juvenile *x* season interaction, group size, year, feeder type, and feeding site. None of the interactions were significant. Feeding increased with increasing group size and was greatest during the post-breeding season (Table 5, Fig 1). The variance estimate for feeding site was 0.017.

**Table 5:** Parameter estimates for generalized linear mixed model predicting the probability of feeding for adult female (≥1.5 years-old) white-tailed deer in Harris County, GA, USA, September–January, 2013–2014.

	*β*	SE	*z* value	Lower CI	Upper CI
**Intercept**	-2.46	0.27	8.94	-2.994	-1.918*
**D**^a^	-0.11	0.16	0.69	-0.425	0.204*
**T**^b^	0.40	0.30	1.33	-0.189	0.991
**Post-breeding**^c^	0.48	0.22	2.18	0.048	0.918*
**Pre-breeding**^c^	0.40	0.21	1.89	-0.015	0.812
**IM**^d^	0.01	0.44	0.03	-0.847	0.876
**J**^e^	0.28	0.24	1.18	-0.187	0.752
**Y**^f^	-0.17	0.19	0.91	-0.544	0.200
**GS**^g^	0.24	0.08	2.87	0.076	0.402*
**FT**^h^^i^	0.88	0.33	2.64	0.226	1.535*
**FT**^h^^j^	0.45	0.29	1.54	-0.124	1.030
**D x T**	0.04	0.10	0.40	-0.155	0.235
**T x Post-breeding**	-0.61	0.43	1.41	-1.456	0.239
**T x Pre-breeding**	-0.40	0.34	1.18	-1.074	0.265
**J x Post-breeding**	-0.52	0.29	1.78	-1.082	0.051
**J x Pre-breeding**	-0.19	0.29	0.64	-0.754	0.382

* Indicates 95% confidence interval does not include zero

^a^ Standardized distance-to-forest

^b^ Time-of-day (1 = diurnal, 0 = nocturnal)

^c^ Compared to reference class: breeding season

^d^ Presence of an immature male

^e^ Presence of a juvenile

^f^ Year

^g^ Group Size

^h^ Feeder type, compared to reference class: barrel feeder

^i^ Feeder type = ground

^j^ Feeder type = trough

Juvenile feeding was best explained by model 4 (Table 2). The best-fitting model included presence of an immature male, presence of an adult female *x* season interaction, group size, year, feeder type, and feeding site. The presence of an adult female *x* season interaction was not significant. Feeding increased with increasing group size and when an adult female was present (Table 6). Juvenile feeding was not influenced by breeding chronology (Table 6, Fig 1). The variance estimate for feeding site was 0.032.

**Table 6:** Parameter estimates for generalized linear mixed model predicting the probability of feeding for juvenile (<1.5 years-old) white-tailed deer in Harris County, GA, USA, September–January, 2013–2014.

	*β*	SE	*z* value	Lower CI	Upper CI
**Intercept**	-1.58	0.25	-6.24	-2.075	-1.083*
**Post-breeding**^a^	-0.34	0.20	1.70	-0.730	0.053
**Pre-breeding**^a^	0.04	0.25	0.15	-0.444	0.518
**MM**^b^	-0.02	0.50	-0.05	-1.007	0.959
**IM**^c^	0.13	0.36	0.37	-0.572	0.834
**AF**^d^	0.66	0.29	2.27	0.090	1.221*
**Y**^e^	-0.56	0.18	-3.12	-0.914	-0.209*
**GS**^f^	0.26	0.13	2.02	0.008	0.520*
**FT**^g^^h^	0.92	0.25	3.63	0.423	1.420*
**FT**^g^^i^	0.39	0.18	2.14	0.032	0.756*
AF*Post-breeding	-0.04	0.28	-0.15	-0.600	0.514
AF*Pre-breeding	-0.35	0.32	-1.11	-0.979	0.273

* Indicates confidence interval does not include zero

^a^ Compared to reference class: breeding season

^b^ Presence of a mature male

^c^ Presence of an immature male

^d^ Presence of an adult female

^e^ Year

^f^ Group Size

^g^ Feeder type, compared to reference class: barrel feeder

^h^ Feeder type = ground

^i^ Feeder type = trough

## Discussion

Our results demonstrate that sex-age class-specific foraging behavior is the result of complex relationships among reproductive chronology, social factors, and predation risk. Individually, these factors have been shown to affect anti-predator behaviors for a wide variety of species, but evidence of how they interact to produce sex-age class-specific foraging behavior is lacking. In the present study, deer appeared to alter foraging behavior in response to socio-sexual factors within the constraints of background predation risk.

We observed marked temporal segregation at feeding sites between mature males and adult females. Several hypotheses have been proposed to explain sexual segregation in sexually-dimorphic ungulates including the predation risk and social factors hypotheses [25]. The predation risk hypothesis posits that sexes segregate due to differential predation risk based on body size, with larger males being less susceptible to predation. According to this hypothesis, males will exploit areas that pose a greater risk of encounters with predators [25]. An alternative hypothesis, the social factors hypothesis, proposes that sexes segregate to avoid aggressive interactions with the opposite sex [25]. In our study, all feeding sites were visited by mature males and adult females making spatial segregation an implausible explanation. It is possible that adult females avoided using the resource when mature males were present to reduce their interactions with behaviorally dominant mature males.

While feeding was similar for all adult sex-age classes during the breeding season, each responded differently to breeding chronology. A breeding male’s strategy for meeting energy-maximizing and time-minimizing requirements [39] during the breeding season may be more effective when utilizing a consistent, high-energy food resource at a feeding site. As with many ungulate species, male white-tailed deer exhibit hypophagia during the breeding season [40,41] and focus time investments in mate searching rather than foraging. Similar to Ozoga and Verme (1982) we noted that adult males visited feeders at lower rates than females and fawns during the breeding season. However, they apparently optimized forage acquisition at these sites by spending a greater proportion of time actively feeding.

During the breeding season, male group size decreases, presumably leading to increased individual predation risk. Our results indicated that mean group size was lowest during the breeding season (1.06 ± 0.01). Additionally, the risk of agonistic encounters with rival males imposes greater social risk during this time. Despite an increase in predation and social risk during the breeding season, mature male feeding did not reflect the change in risk.

Immature male feeding did not differ from the pre-breeding to breeding season but was reduced during the post-breeding season. Decreased feeding by immature males during the post-breeding season may have resulted from conspecific-directed vigilance [14,22]. Competition and social rank have been used to explain alterations of vigilance levels in ungulates including impala [42], elk [14], Przewalski's gazelle [15], and Père David’s deer [22]. Although we could not obtain convergence for our immature male model when including a season and mature male interaction, we observed 1.9*x* as many photographs per week of mature males and immature males together during post-breeding season than during the pre-breeding season. Consequently, immature males may have increased vigilance toward mature males during the post-breeding season.

Juveniles were more likely to be feeding than adults during all seasons. Juveniles are generally less wary than adults despite typically being the most vulnerable demographic. Also, because juveniles typically do not reach sexual maturity during their first year in the southeastern U.S., their activity budget focuses more on body growth [13] than reproduction. We detected an increase in juvenile feeding when adult females were present but, interestingly, adult female feeding was not affected by the presence of a juvenile.

We predicted that adult females would feed least during the pre-breeding season when their fawns were more susceptible to predation as the survival of their offspring determines their lifetime fitness [43]. However, our results did not support this prediction. According to Williams (1966), a parent should maximize lifetime fitness by balancing investment in present and future reproduction. In our study, juveniles were likely weaned by the beginning of data collection. Because dams should base their investment decisions on the value of their current offspring relative to future reproduction [44], adult females may invest more heavily in future reproduction via their own survival (i.e. not increasing vigilance at the cost of decreasing their own fitness through reduced resource acquisition rates) than in the survival of the juvenile. Cherry et al. (2015) suggested that there is a point at which females must allocate more energy to personal nutrition to foster gestation than to protection of their offspring.

Distance to escape cover is an important consideration for animals when making foraging decisions [9,45]. Animals perceive predation risk at multiple spatial scales and alter anti-predator behavior in response to the perceived predation risk associated with specific locations [10]. Lagory (1989) found that white-tailed deer vigilance decreased in more open areas. However, Lagory (1986) investigated this relationship on an island where predators had been extirpated for many years. To the contrary, we hypothesized that feeding would decrease with increasing distance-to-forest because, in general, cover benefits prey with cursorial predators. We found that feeding decreased with increasing distance-to-forest for mature males only.

Each sex-age class increased feeding with increasing group size. Previous studies on white-tailed deer vigilance demonstrated that males and females decreased vigilance when foraging in larger groups [7,27,33]. Cherry et al. (2015), however, reported an increase in proportion of time spent feeding with increasing group size for females and juveniles but not males. They attributed the lack of response by males to increasing group size to intra-specific competition at a concentrated resource and timing of sampling relative to breeding chronology. Our research investigated group size-feeding relationships at a broad temporal scale (September–January) and, therefore, may not have detected finer temporal scale responses by males.

How prey animals alter vigilance in response to changes in breeding chronology, social dynamics, environmental, and habitat-related factors is important for understanding underlying drivers of ecological processes. Behavioral modifications induced by predation risk can have cascading effects on plant and animal communities [46,47], especially in systems characterized by abundant herbivore populations. In addition to balancing the tradeoffs between resource acquisition and predator avoidance, animals must also alter behavior in accordance with the cost-benefits of vigilance directed at conspecifics [14]. Our results suggest that within a single species, each sex-age class shows differential responses to socio-sexual factors within the constraints of predation risk. Given the recent changes in the distribution and abundance of non-human predators to landscapes that many prey populations inhabit [48–50], future research comparing the relative influences of socio-sexual and predation risk on vigilance behaviors are warranted.

## Supporting information

S1 Dataset (ZIP)
